# Local Network Properties of Soil and Rhizosphere Microbial Communities in Potato Plantations Treated with a Biological Product Are Important Predictors of Crop Yield

**DOI:** 10.1128/mSphere.00130-21

**Published:** 2021-08-11

**Authors:** Nabeel Imam, Ignacio Belda, Beatriz García-Jiménez, Adrian J. Duehl, James R. Doroghazi, Daniel E. Almonacid, Varghese P. Thomas, Alberto Acedo

**Affiliations:** a Biome Makers Inc., West Sacramento, California, USA; b Department of Genetics, Physiology and Microbiology, Complutense University of Madrid, Madrid, Spain; c Bayer U.S., Chesterfield, Missouri, USA; d Bayer U.S., Cary, North Carolina, USA; e Bayer U.S., West Sacramento, California, USA; National Institute of Advanced Industrial Science and Technology

**Keywords:** agricultural biological, machine learning, soil microbiome, yield prediction

## Abstract

Understanding the effectiveness and potential mechanism of action of agricultural biological products under different soil profiles and crops will allow more precise product recommendations based on local conditions and will ultimately result in increased crop yield. This study aimed to use bulk soil and rhizosphere microbial composition and structure to evaluate the potential effect of a Bacillus amyloliquefaciens inoculant (strain QST713) on potatoes and to explore its relationship with crop yield. We implemented next-generation sequencing (NGS) and bioinformatics approaches to assess the bacterial and fungal biodiversity in 185 soil samples, distributed over four different time points—from planting to harvest—from three different geographical locations in the United States. In addition to location and sampling time (which includes the difference between bulk soil and rhizosphere) as the main variables defining the microbiome composition, the microbial inoculant applied as a treatment also had a small but significant effect in fungal communities and a marginally significant effect in bacterial communities. However, treatment preserved the native communities without causing a detectable long-lasting effect on the alpha- and beta-diversity patterns after harvest. Using information about the application of the microbial inoculant and considering microbiome composition and structure data, we were able to train a Random Forest model to estimate if a bulk soil or rhizosphere sample came from a low- or high-yield block with relatively high accuracy (84.6%), concluding that the structure of fungal communities gives us more information as an estimator of potato yield than the structure of bacterial communities.

**IMPORTANCE** Our results reinforce the notion that each cultivar on each location recruits a unique microbial community and that these communities are modulated by the vegetative growth stage of the plant. Moreover, inoculation of a Bacillus amyloliquefaciens strain QST713-based product on potatoes also changed the abundance of specific taxonomic groups and the structure of local networks in those locations where the product caused an increase in the yield. The data obtained, from in-field assays, allowed training a predictive model to estimate the yield of a certain block, identifying microbiome variables—especially those related to microbial community structure—even with a higher predictive power than the geographical location of the block (that is, the principal determinant of microbial beta-diversity). The methods described here can be replicated to fit new models in any other crop and to evaluate the effect of any agricultural input in the composition and structure of the soil microbiome.

## INTRODUCTION

Potato, the stem tuber vegetable produced by Solanum tuberosum, is the crop with the highest yield out of the five most important agricultural crops in the world (rice, wheat, soybeans, maize, and potatoes). Although global production of potatoes in 2012 reached 364,808,768 metric tons, it has been calculated that actual yield corresponds to only about 10% to 75% of potential yield (1). Improving global agricultural crop production in a sustainable way is paramount given the current prospects for world population increase (2).

Potato yield has been directly correlated with edaphological and climate variation (3–5), with management practices (6), and with potato cultivar (7). Interestingly, the same biogeographical patterns have been identified as the main drivers of microbial community composition in potato plants (8–15), reinforcing the key role of soil microbiology in potato crop productivity (16). Thus, in agroecosystems, the enhancement and sustainability of productivity can be assessed by means of the soil microbiome. Additionally, the Natural Resources Conservation Service of the US Department of Agriculture (17) links soil quality with the concept of soil health, acknowledging the relevance of soil microorganisms to drive soil functionality.

In this context, the use of substances, microorganisms, or mixtures thereof, known as plant biostimulants, is among the latest practices for sustainable food and energy production (18). Biological products are claimed to promote plant health and quality and recycling crop residues with low environmental impact (19, 20). Not surprisingly, the market for agricultural biological products is recording a compound annual growth rate (CAGR) of over 10% since 2017, and it is expected to reach a market size of over 4 billion dollars by 2025 (21). Rajabi-Hamedani et al. (22) argue that this growth is a consequence of the need to increase the efficiency of agrochemical inputs, to reduce crop damage caused by abiotic stress, and to reduce the environmental impact of production systems.

Most agricultural biological products based on microorganisms are expected to pertain to the functional group of Plant Growth Promoter species, so a direct impact in plant health (23, 24) and yield (25) is assumed. Different direct mechanisms involved in yield promotion have been demonstrated in certain bacterial strains, including (i) improving growth of tomato plants, by increasing root hair development in a phytohormone-mediated process using an Azospirillum brasilense strain (26) or by increasing the tolerance to abiotic stresses through the action of a 1-aminocyclopropane-1-carboxylate (ACC) deaminase produced by a Burkholderia unamae strain (27), (ii) increasing plant growth by enhanced nutrient (P) acquisition in cucumber and tomato plants using a *Bacillus* sp. strain (28), (iii) enhancing nodule formation by a two-species consortium of Pseudomonas putida plus *Rhizobium* sp. in beans (29), or by improving grain yield in rice by increasing panicle number through the use of an Azospirillum amazonense strain (30). In addition, some microbial strains have also shown an indirect effect in soil and plant health, as tools for *in situ* microbiome engineering, promoting the development of other beneficial microbial species, improving the resistance of the microbiome to the invasion of plant pathogens, and increasing the natural resistance of the plant against diseases (31).

Instead of assuming a simple, unidirectional and direct effect of a certain microbial strain in the physiology and development of plants, agricultural biological products face challenges with consistent field performance. Different strains and species can have different functional performance under specific environmental and ecological conditions (32). For this reason, biological products’ claims need to describe ecological and functional performance and not only be based on composition of matter (33).

In this work, we aimed to contribute to global sustainability of the agricultural lands by demonstrating that assessments of bulk soil and rhizosphere microbial composition and structure can be practical tools to substantiate agricultural biological product claims, and at the same time they provide a toolkit for growers to assess and achieve increased yield and sustainability of their management practices. Applying “-omics” technologies, we explored the subtle side effects of the microbial inoculant Bacillus amyloliquefaciens strain QST713, in the surrounding rhizosphere and bulk soil microbiota of potatoes, and its potential connection with the yield observed. We followed the recommendations of Ricci et al. (33) for field trials in one crop, in order to demonstrate that this product has bona fide effects. We were particularly interested in comparing the microbiome profile associated with treated versus untreated samples over time and across diverse locations, to determine whether or not a common mechanism of action was at play. Both the changes in the microorganism composition of samples across time and the evolution of the structure of the bacterial and fungal communities were assessed. Additionally, making use of potential correlations among microbiome profiles, product use, and crop yield, we built a yield prediction model as a first step toward guaranteeing growers the level of effectiveness of a product under different management and environmental conditions (weather, soil microbiome, soil type, seed variety, etc.). Our observations conclude that individual microorganism abundances as well as the structure of the fungal and bacterial communities change slightly after application of the inoculant but that these changes are more evident in those locations responding with an increased yield to the application of the product.

## RESULTS

**FIG 1:**
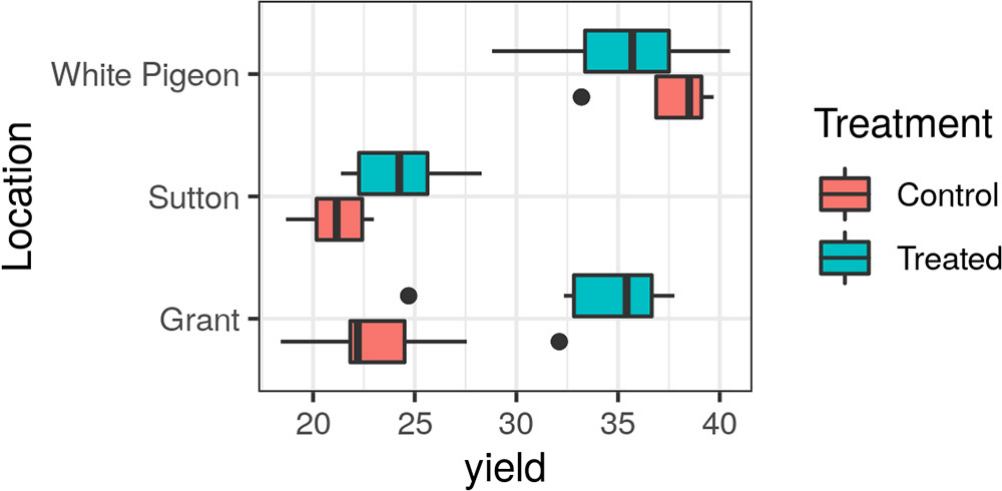
Yield data (t/ha) for control and treated blocks across locations. Yield = 30 separates blocks into two categorical variables (≤30 t/ha, >30 t/ha) and corresponds to one of the natural zero-probability density points in the bimodal yield distribution. The box limits correspond to the 25th and 75th percentiles, and the central line is the median. The whiskers are the 5th and 95th percentiles. The dots represent outliers [points below 25th percentile − (1.5 × IQR) and above 75th percentile + (1.5 × IQR), where IQR is the interquartile range or absolute difference between 75th and 25th percentiles].

In this work, we assessed bacterial and fungal communities of bulk soil and rhizosphere of potato plantations from three different locations of the United States: Sutton and Grant (Idaho) and White Pigeon (Michigan). Our main aim was to understand the effect of a microbial inoculant (*B. amyloliquefaciens* strain QST713) on the rhizosphere microbiota and its final legacy in the bulk soil microbiota after harvest. We were also trying to identify potential microbiome biomarkers associated with plantation blocks with low or high yields. A total of 185 samples from treated and untreated blocks at each location were collected over four time points, from planting (T0) to harvest (T3), focusing on the early changes occurring after 1 (T1) and 2 (T2) months from planting, where T0 and T3 are bulk soil samples and T1 and T2 are rhizosphere samples. Figure 1 shows that, in two of the three locations assayed, the use of the inoculant had a significant effect on increasing the crop yield (Grant *P* value 8.23 × 10^−10^ and Sutton *P* value 7.41 × 10^−7^), without any detectable effect in the third location (White Pigeon *P* value 0.30), which had, indeed, a much higher yield in both control and treatment samples.

### Geographical location and sampling time determine the microbiome composition of potato cultivars.

**FIG 2:**
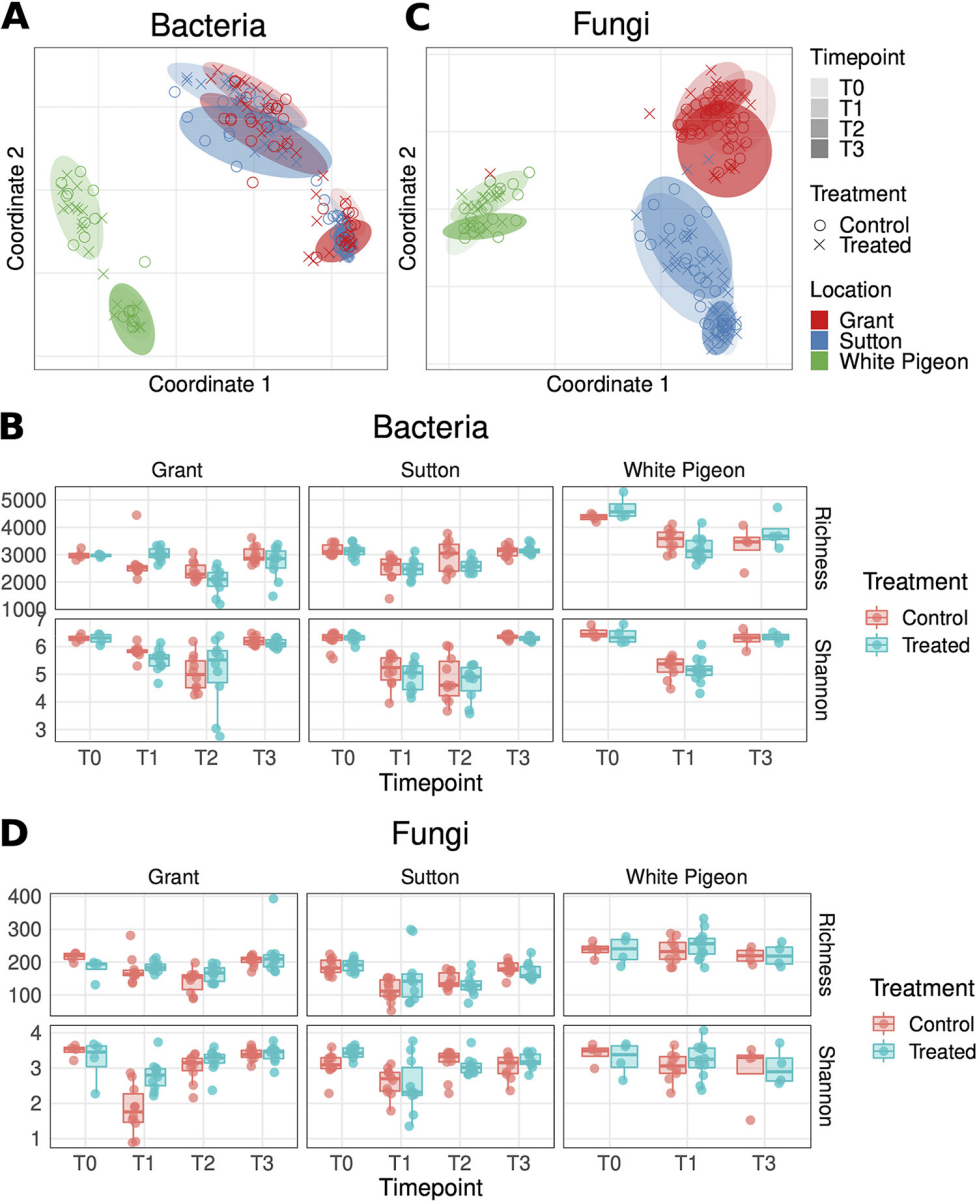
Beta- and alpha-diversity of bacterial and fungal populations in samples across locations and sampling times. (A and C) Beta-diversity (PCoA ordination) of bacterial and fungal populations. (B and D) Alpha-diversity (OTU richness and Shannon [H′] index) of bacterial and fungal populations. T0, before planting; T1, 1 month after planting; T2, 2 months after planting; T3, after harvesting. Box plot limits are the same as defined for Fig. 1.

Figure 2 shows a clear population pattern differentiating bulk soil samples (T0 and T3; before planting and after harvesting, respectively) and rhizosphere samples (T1 and T2 samples; 1 and 2 months after planting, respectively) in all locations, with a clearer pattern in bacteria than in fungi. Figure 2A shows that in terms of beta-diversity of bacterial populations, location (*R*^2^ = 0.353, *P* value 0.001) and sampling time (*R*^2^ = 0.292, *P* value 0.001) had significant effects, with the treatment (*R*^2^ = 0.004, *P* value 0.07) having a minor marginally significant effect. However, for fungal populations (Fig. 2C), location dominates as the main driver of the beta-diversity patterns (*R*^2^ = 0.379, *P* value 0.001), with sampling time having a much lower impact (*R*^2^ = 0.089, *P* value 0.001) than in bacterial populations, but with treatment also having a small but significant (*R*^2^ = 0.007, *P* value 0.044) impact (see full permutational multivariate analysis of variance [PERMANOVA] data in Table S1 in the supplemental material). As shown in Fig. 2A and C, White Pigeon presents beta-diversity patterns significantly different from Grant and Sutton; this can be easily explained by the geographical distance between locations, which correlates well with the Aitchison distances of samples in the principal-coordinate analysis (PCoA). In addition, it should be stated that, unfortunately, in this work it is hard to dissect the contribution of seed variety from that of geography, since we do not have different seed varieties planted within the same location but only the same seed variety in the closely located sites of Grant and Sutton (Idaho) and a different one in White Pigeon (Michigan). Thus, although in this work we have focused our discussion on the effect of geographical location, the reader should be conscious that geographical location (*R*^2^ = 0.353 for bacteria; *R*^2^ = 0.379 for fungi), seed variety (*R*^2^ = 0.310 for bacteria; *R*^2^ = 0.312 for fungi), and their interaction (location:variety) (*R*^2^ = 0.332 for bacteria; *R*^2^ = 0.349 for fungi) have similar estimate values (*P* value 0.001 in all cases) as explaining variables of beta-diversity (Table S1). There are also different edaphological and weather conditions at each of these locations, and a different seed variety in White Pigeon compared to Sutton and Grant, all of which are major drivers of the soil microbial populations as previously observed by Rasche et al. (10) and İnceoğlu et al. (14) in potato soils. The significant differences between microbial community compositions before and after planting can be clearly seen at Fig. 2A and C, where, despite the large differences between locations, T1 and T2 samples clustered in all the three locations, away from their respective T0, especially in the case of bacterial populations. Similar observations have been reported in maize (34), rice (35), and potato cultivars (13), and in forest soils (36).

10.1128/mSphere.00130-21.3 TABLE S1 PERMANOVA results for bacterial and fungal beta-diversity considering (i) location, time point, variety, and treatment as covariates (Full_PERMANOVA_16S and Full_PERMANOVA_ITS); (ii) interaction location:variety (Location:Variety_PERMANOVA_16S and Location:Variety_PERMANOVA_ITS); and (iii) separate analysis of location (Location_PERMANOVA_16S and Location_PERMANOVA_ITS) and variety (variety_PERMANOVA_16S and variety_PERMANOVA_ITS). Download Table S1, XLSX file, 0.04 MB. Copyright © 2021 Imam et al. 2021 Imam et al. https://creativecommons.org/licenses/by/4.0/ This content is distributed under the terms of the Creative Commons Attribution 4.0 International license.

Regarding alpha-diversity (Fig. 2B and D), there is a clear impact of planting in reducing the diversity of bacterial and fungal populations across locations (Table S2; see results with the label “all,” for global data normalized [Z-score] per location), as shown in T0-to-T1 and T0-to-T2 comparisons (in both operational taxonomic unit [OTU] richness and Shannon [H′] index values) for bacterial populations and in T0-to-T1 (Shannon index) and T0-to-T1 and T0-to-T2 (OTU richness) comparisons in fungal populations. This trend suggests that the early stages of the assembly of the rhizosphere microbiota reduce the microbial alpha-diversity previously found in the bulk soil before planting. When soil samples were again analyzed after harvest (T3) in all the locations, we observed that in spite of the marked microbial succession patterns found from T0 to T2, there were no significant changes in alpha-diversity between the microbial communities found in the soil before planting (T0) and after harvesting (T3) (Table S2); therefore, in spite of the changes observed during the assembly of the rhizosphere community, there is not a persistent legacy in the microbial communities of the bulk soil (sampled at the same place where the plant used to be) after harvesting. Additionally, comparing control and treated samples at the same time point, we observed significant changes only in Grant at T1 for bacterial OTU richness and Shannon index as well as fungal Shannon index (Table S3). Interestingly, Grant was the site with the largest yield increase response due to treatment. However, it should be noted that, although additional studies are necessary (ideally by strain-targeted qPCR) to monitor the persistence of the inoculated *B. amyloliquefaciens* strain in the rhizosphere and bulk soil after harvesting, we did not detect any significant increase in the *Bacillus* populations at any sampling time (evolution from T0 to T1, T2, and T3) in treated versus untreated blocks (Table S4).

10.1128/mSphere.00130-21.4 TABLE S2 Results of ANOVA comparing the evolution of alpha-diversity (OTU richness and Shannon [H′] index) of bacterial and fungal populations from T0 to the remaining time points (T1, T2, and T3). Download Table S2, XLSX file, 0.01 MB. Copyright © 2021 Imam et al. 2021 Imam et al. https://creativecommons.org/licenses/by/4.0/ This content is distributed under the terms of the Creative Commons Attribution 4.0 International license.

10.1128/mSphere.00130-21.5 TABLE S3 Wilcoxon rank sum test for bacterial and fungal alpha-diversity of control versus treated samples. Download Table S3, XLSX file, 0.01 MB. Copyright © 2021 Imam et al. 2021 Imam et al. https://creativecommons.org/licenses/by/4.0/ This content is distributed under the terms of the Creative Commons Attribution 4.0 International license.

10.1128/mSphere.00130-21.6 TABLE S4 Significant differential abundance of bacterial and fungal OTUs of control versus treated samples at T1 versus T0 and T2 versus T0. Download Table S4, XLSX file, 0.02 MB. Copyright © 2021 Imam et al. 2021 Imam et al. https://creativecommons.org/licenses/by/4.0/ This content is distributed under the terms of the Creative Commons Attribution 4.0 International license.

At the taxonomy level, despite clear population dynamic patterns from T0 to T2 sampling times in all the three locations and in both treated and untreated samples, samples from all three locations and times shared some of the most abundant genera for both bacterial and fungal communities (Fig. S1). Figure S1 shows the top bacterial genera identified across samples in this study (core microbial genera). Of these, five (*Arthrobacter*, Pseudomonas, *Sphingomonas*, *Streptomyces*, and *Rhizobium*) also appeared in the soil bacterial survey performed by İnceoğlu et al. (13) on potato fields. Among the top fungal genera shared across samples in our study (core fungal genera), we found Cryptococcus, *Mortierella*, and *Alternaria*, which, as far as we know, have not been previously described as part of the core soil and rhizosphere microbiota of potato plants but are, indeed, some ubiquitous fungal genera in agriculture soils.

10.1128/mSphere.00130-21.1 FIG S1 Taxonomic composition of soil samples across locations and sampling times. (A) Most abundant bacterial genera identified. (B) Most abundant fungal genera identified. T0 - before planting; T1, 1 month after planting; T2, 2 months after planting; T3, after harvest. Download FIG S1, TIF file, 0.4 MB. Copyright © 2021 Imam et al. 2021 Imam et al. https://creativecommons.org/licenses/by/4.0/ This content is distributed under the terms of the Creative Commons Attribution 4.0 International license.

However, as previously reported (37) in tomato cultivars using a Bacillus subtilis strain, and in soybean (38) and lettuce (39) cultivars using different strains of *B. amyloliquefaciens*, here we did not detect a durable impact of the treatment on the bulk soil microbial communities after harvesting (T3) in terms of major taxa (Fig. S1) and alpha- and beta-diversity (Fig. 2; see also Tables S1 and S2).

### Elements of microbiome composition and structure can be modulated by use of a *B. amyloliquefaciens*-based soil applied biological product.

To dissect the specific effect of the biological product over the microbial composition across time at each location, we compared the fold change of each OTU in the treatment group from T0 to T1 (and from T0 to T2) with the fold change in the control group at the same time intervals per location (Table S4). Out of 17,241 unique bacterial OTUs in the samples of the study, 16 changed significantly from T0 to T1 (detected just in White Pigeon), and 85 from T0 to T2 (14 in Grant and 71 in Sutton). These OTUs belong to 68 genera, of which 5 changed significantly in at least two locations and in the same direction (increase or decrease) in all locations: *Clostridium*, *Sphingopyxis*, and *Woodsholea in* Grant and Sutton and *Flavobacterium* and *Pedobacter* in Sutton and White Pigeon. For fungi, out of 1,702 unique OTUs, 10 OTUs changed significantly from T0 to T1 (none in Grant, seven in Sutton, and three in White Pigeon), and 31 from T0 to T2 (none in Grant, 31 in Sutton, and none in White Pigeon). These OTUs belong to 29 genera, of which none changed significantly, in the same direction (increase or decrease), in at least two locations. In summary, despite location and sampling time having a larger effect than treatment on the composition of microorganism populations, the inoculant still generated common detectable abundance changes in at least two of the three locations for several bacterial genera, some of which have known functionally relevant roles (i.e., *Flavobacterium* and *Pedobacter*).

In order to get a deeper understanding of how the structure of the bacterial and fungal communities, and therefore the ecological relationships among microorganisms, impacts the effect of the bacterial inoculant, we studied the cooccurrence and coexclusion patterns between pairs of OTUs in each sample of the trial. As some of us reported in a recent work (40), by studying the network properties of local communities inferred from the cooccurrences and coexclusion patterns of a reference metacommunity it is possible to estimate ecological emergent properties (i.e., niche specialization, level of competition) of interest for the understanding of microbiome functioning (41). We first built metacommunities based on all samples of the trial and then explored the structure of local microbiome communities, based on just the nodes present in each individual sample, aiming to detect changes in network properties that are associated with the application of the biological product at a specific location over time. Specifically, for the coexclusion and cooccurrence bacterial networks, we calculated the modularity (a measure of the strength of partitioning of a network into modules) and transitivity (measure of the degree to which nodes in a network cluster together) as well as the proportion of coexclusions and cooccurrences present in the local network compared to the total number of possible combinations among all OTUs in the sample.

**FIG 3:**
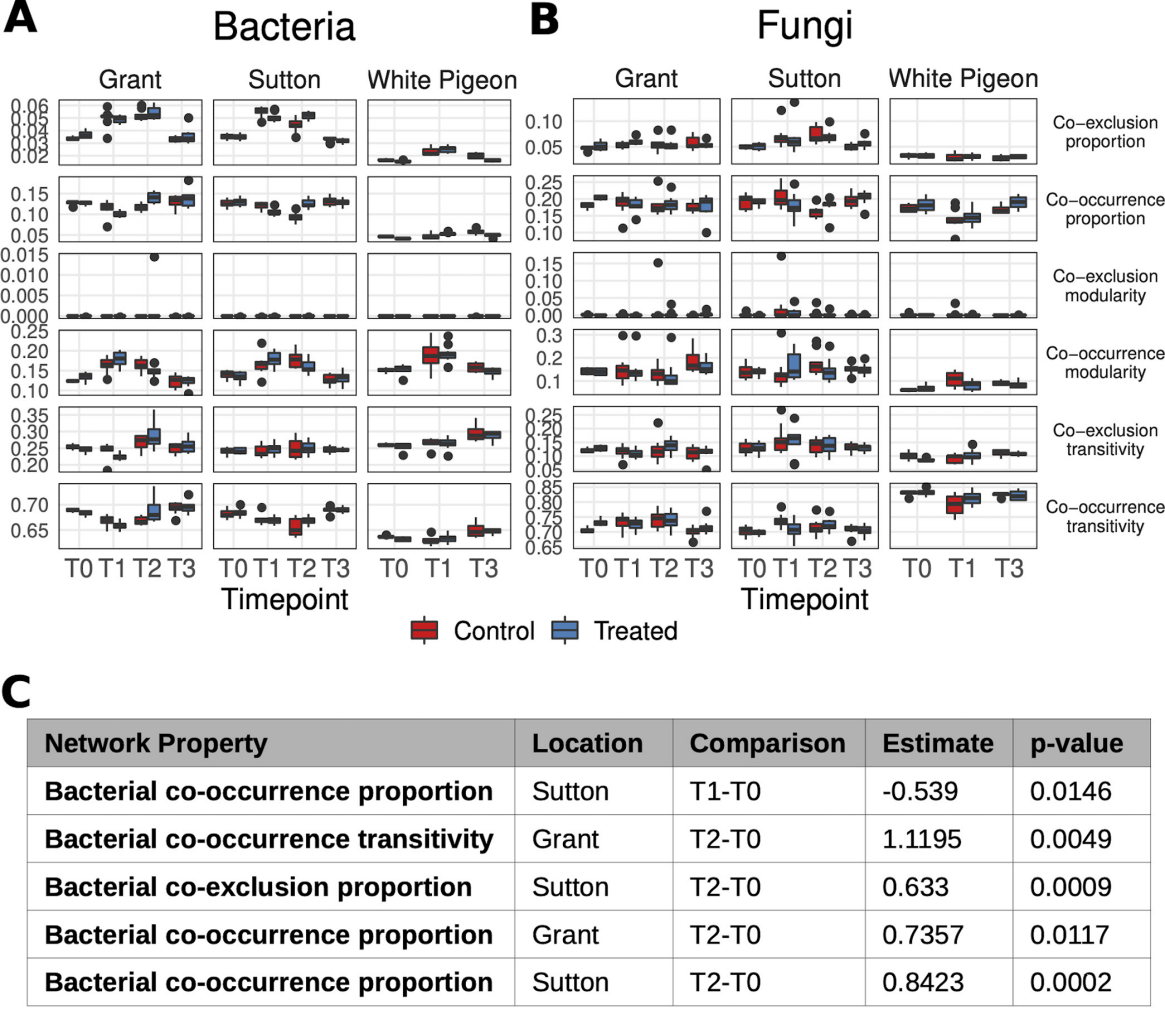
Local network properties across locations and sampling times. (A and B) Local network properties of bacterial and fungal populations in samples from the three locations (Grant, Sutton, and White Pigeon) at all sampling times (from T0 to T3). (C) Significant changes from T0 to T1 and from T0 to T2 in treated versus untreated blocks (no significant changes were detected from T0 to T3).

Figure 3A and B show the evolution from T0 to T3 of the six local network properties studied across locations, for bacterial and fungal populations, respectively. Figure 3C lists those changes that have been significantly different in time—from T0 to T1, T2, and T3—in treated versus untreated blocks (see Table S5 for full data). In Grant, there are significantly higher changes in bacterial cooccurrence proportion and transitivity values from T0 to T2 in the treated samples compared to untreated ones. In Sutton, both the bacterial cooccurrence and coexclusion proportion changed differently in time in treated blocks in comparison with untreated blocks. Thus, when attending to the microbiome structure changes caused by the treatment, we detected significant changes just in Grant and Sutton, which were the locations where treatment had a significant effect over yield, but no significant changes caused by the treatment in the evolution of network properties in White Pigeon. The detected changes in Grant and Sutton occurred from T0 to T1 and/or T2, but no significant changes were detected from T0 to T3.

10.1128/mSphere.00130-21.7 TABLE S5 Network property changes of bacterial and fungal communities of control versus treated samples at T1 versus T0 and T2 versus T0. Download Table S5, XLSX file, 0.01 MB. Copyright © 2021 Imam et al. 2021 Imam et al. https://creativecommons.org/licenses/by/4.0/ This content is distributed under the terms of the Creative Commons Attribution 4.0 International license.

### Elements of microbiome composition and structure allow prediction of potato yield.

**FIG 4:**
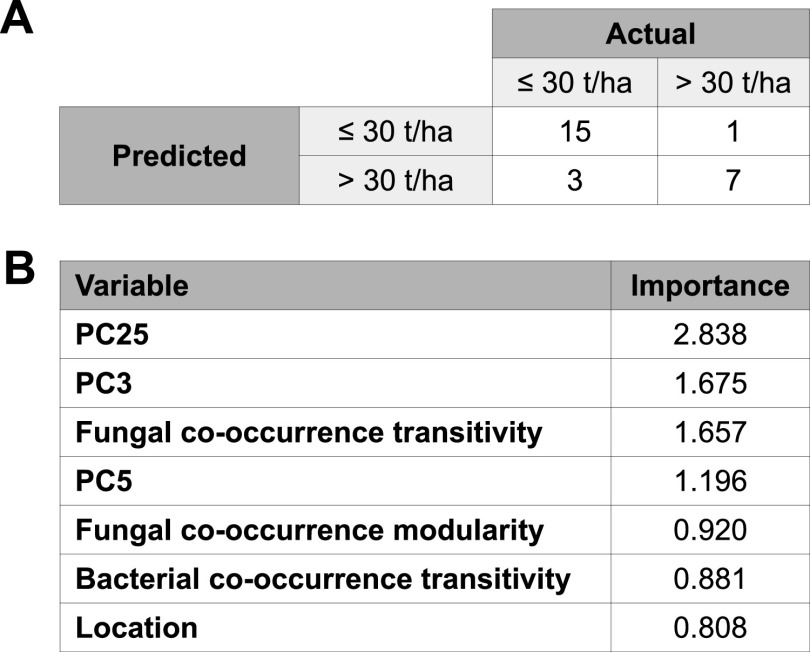
Random Forest yield model fitted to predict blocks with yields of ≤30 t/ha or >30 t/ha based on soil microbiome composition and structure data. (A) Confusion matrix for the Random Forest model over the test set samples. (B) Importance figures of the main variables contributing to the predictive power of the Random Forest model.

We fitted a Random Forest model aiming to predict if a rhizosphere or bulk soil sample comes from a block with a yield of ≤30 tons/hectare (t/ha) or >30 t/ha, based on its microbiome composition and structure using multivariate compositional data (principal components from a beta-diversity ordination) and local network properties. We measured yield data in 20 treated and 20 untreated blocks from the three geographical locations, and for each we utilized all samples available over times T0, T1, and T2. In total, 104 samples were used for this task, split into a training set of 78 samples and a test set of 26 samples. The result of this model showed a predictive accuracy of 84.6% (Fig. 4A) and identified six variables (three network properties and three compositional) as the most important predictors of yield (Fig. 4B), even with a higher importance than a variable that we used to encompass the effects of geography that is not accounted for by the microbial composition and structure. Surprisingly, although we detected only some treatment-mediated changes in the temporal dynamics of bacterial network properties (Fig. 3C), the structure of fungal communities—in particular, the fungal cooccurrence transitivity and modularity—accounted for a higher importance as predictors of yield than any bacterial network property (Fig. 4B; see Table S6 for full data on the importance of variables to the yield prediction model). In particular, we confirmed that there is a positive correlation between the cooccurrence transitivity of bulk and rhizosphere soil fungal communities and the yield found in the different blocks (*R*^2^ = 0.32, *P* value 0.05). This is a particularly important observation for understanding the effect of the *B. amyloliquefaciens*-based biological product assayed here in shaping the structure of fungal communities as a potential mechanism of action in those places where it facilitates an increase in the yield.

10.1128/mSphere.00130-21.8 TABLE S6 Importance of variables in Random Forest yield predictive models. Download Table S6, XLSX file, 0.01 MB. Copyright © 2021 Imam et al. 2021 Imam et al. https://creativecommons.org/licenses/by/4.0/ This content is distributed under the terms of the Creative Commons Attribution 4.0 International license.

The other highlighted compositional variables (PC25, PC3, and PC5) contributing to the predictive power of the model fitted can be explored by looking at the taxonomy of the OTUs in each showing a significant correlation with the yield. It is necessary to keep in mind that the predictive power of PC variables, as principal components of a multivariate analysis, came from the interaction patterns among the OTUs and not from their individual behavior. However, we can highlight the presence, for instance, of the bacterial biocontrol-related genus *Serratia* (42) as the taxonomic identity of the OTU with the highest positive correlation with yield in PC25 and *Bacillus* sp. as the taxonomic identity of the OTU with the highest positive correlation with yield in PC3 (Fig. S2). Although we cannot confirm that the detected OTU pertains to the inoculated *B. amyloliquefaciens* strain, it is interesting to find this significant correlation of a *Bacillus* OTU with yield.

10.1128/mSphere.00130-21.2 FIG S2 Taxonomic assignments and their relationship with yield (fold change values) of the OTUs contributing to the 10 most important principal components of the beta-diversity ordination generated for the yield predictive model. Download FIG S2, TIF file, 0.5 MB. Copyright © 2021 Imam et al. 2021 Imam et al. https://creativecommons.org/licenses/by/4.0/ This content is distributed under the terms of the Creative Commons Attribution 4.0 International license.

As described in Materials and Methods, since yield is constant for all samples within a block, we converted yield to a categorical variable (≤30 t/ha, >30 t/ha). The distribution of the yield data was bimodal, and thus, it seemed logical to divide the categories on a zero-probability density point for the bimodal distribution. However, in order to assess if this decision may have had an impact in the features identified as important by the yield predictive model presented here, we investigated the models resulting from splitting the yield data into three (≤26 t/ha, >26 t/ha to ≤35 t/ha, and >35 t/ha) or four (≤20 t/ha, >20 t/ha to ≤26 t/ha; >26 t/ha to ≤35t/ha, and >35 t/ha) categories. As can be seen from Table S6, fungal cooccurrence transitivity always had higher importance than geographical location and seed variety, independently of the number of yield categories used. However, dividing yield into more categories resulted in decreased accuracy (65.4% when splitting into three categories and 61.5% when splitting into four categories) due to the limited training set size being divided into an increasing number of categories.

## DISCUSSION

The use of microbial inoculants to increase the yield of plants is a useful strategy, increasingly used in agriculture. In addition to the direct impact of the microbial inoculant in the plant, due to its unique metabolic properties, the introduction of an allochthonous strain in the microbial rhizosphere and bulk soil ecosystems may have an impact on the entire microbiome, affecting the composition and structure of the native communities. Our work demonstrates that geographical location being the main driver of the microbial profile of rhizosphere and bulk soils from potatoes, sampling time (including different sample types [bulk soil and rhizosphere] and phenological stages of the plant) also has a major impact on the microbiome composition, especially in the bacterial community. Relegated to last position, the use of a microbial inoculant based on *B. amyloliquefaciens* QST713—a strain isolated from the soil of a Californian organic peach orchard with a demonstrated effective broad-spectrum bactericide and fungicide activity (43) through a number of different mechanisms of action (44, 45)—showed a slight but significant effect on the beta-diversity of fungal communities (*R*^2^ = 0.007, *P* value 0.04) and an even slighter and just marginally significant effect on bacterial communities (*R*^2^ = 0.004, *P* value 0.07) (see Table S1 in the supplemental material). Given that geographical location and sampling time have such strong influence over bulk and rhizosphere soil community composition and structure, the treatment effects observed were analyzed per location as evolution between two time points when comparing treated with untreated blocks. This also means that the patterns identified here as derived from product use may be of a more correlative than deterministic nature. Nonetheless, the abundance of several bacterial genera changed significantly, and consistently in at least two locations, from T0 to T1 and from T0 to T2 in the treated blocks, including several functionally important members of the soil microbiota, as well as modifying the evolution in time of specific bacterial network properties. Importantly, we also observed that this *B. amyloliquefaciens* QST713 trial did not cause any persistent legacy effect on the microbiome profile of the soil analyzed after harvesting, i.e., the effect of the bioinoculant is limited in time and the native microbiome returns to its original state after harvesting.

We also presented here a machine learning potato yield prediction model, based on a soil health assessment of its microbial composition and structure. Van Klompenburg et al. (46) performed a systematic literature review to identify the most used machine learning algorithms for crop yield prediction as well as the most used features to train those algorithms. They identified that most researchers have used neural networks in their work with the most frequently used features being temperature, rainfall, and soil type. Interestingly, none of the articles reviewed utilized soil microbial or fungal composition or structure as features. In recent work, Jeanne et al. (16) developed a model to correlate potato yield with soil bacterial diversity. They showed that their species balance index related to potato yield (SBI-py) had a high correlation (0.77) with yield, whereas the Shannon diversity, Pielou diversity, and Chao 1 diversity failed to correlate well with yield. Our Random Forest model can predict with relatively high accuracy whether a potato block will have a yield of more or less than 30 t/ha, which was the value that divided the bimodal distribution of yield in our training set. This model is our first step toward understanding not only why but also when and where biological products work to increase yield. It is also worth noting that the data set in this study included as a variable the application of a bioinoculant; thus, this yield model also represents a first step toward understanding when and where biological products work. Despite the small sample size and the treatment of yield as a categorical value, independent of the number of categories used for splitting yield, we always found that the structure of fungal rather than bacterial communities was a better potato yield estimator (for instance, fungal cooccurrence network transitivity), which has not been reported before and merits further study.

In addition, the significant contribution of the local network properties to the estimation of the actual yield of a certain block reinforces the idea of the need of a more functional vision of agriculture microbiomes, as certain emergent properties can be deduced from them. In this context, *B. amyloliquefaciens* QST713 caused some changes in the evolution of the structure of bacterial rhizosphere microbiota, so it is also interesting to include this network-based approach in future studies on the mechanism of action of bioinoculants in crops.

Our model trained in only three locations, including only two potato varieties, and where half of the samples were treated with the bioinoculant, may be biased, for instance, in recognizing the effects of *B. amyloliquefaciens* QST713 over the soil microbiome as the main features predictive of yield. Possible future avenues of research derived from the current work include (i) monitoring the persistence of the inoculant in the rhizosphere, in the short term, and in the bulk soil, in the long term, and its effect on the abundance of bacterial and fungal communities but also investigating its potential colonization of endophytic sites and a potential direct plant growth-promoting activity; (ii) investigating whether fungal cooccurrence network transitivity continues to be an important variable in models predicting yield in a more diverse data set than the one described here (more varieties, more locations, more samples, wider variety of edaphological and weather conditions); (iii) building predictive models containing not only microbiome data but also edaphological and climate information; and (iv) investigating the link between local network properties of microbial communities and ecosystem multifunctionality and their ecological meaning as potential indicators of niche differentiation and competition; among others. Ultimately, the positive correlation between crop yield and fungal cooccurrence transitivity identified in this study is a useful concept to design and test interventions for increasing crop yield and should be further studied as a potential mechanism of action of *B. amyloliquefaciens* QST713 in those cases where it increases the yield of potato cultivars. This finding also demonstrates that assessments of soil microbial composition and structure in agricultural input trials can be practical tools to substantiate biological product claims and that they provide a toolkit for growers to assess and achieve increased yield and sustainability of their management practices.

## MATERIALS AND METHODS

### Field trials.

Russet Burbank potatoes were planted in the Sutton (43.7963867, −116.927742) and Grant (43.7552948, −116.815269) locations (Idaho, USA). Seed variety Lamoka was planted in White Pigeon (41.7776489, −85.6745224) (Michigan, USA). Applications were performed at 46.770 liters/ha of spray volume combining grower standard practice of Quadris and Admire Pro plus the biological treatment, tank mixed and applied in-furrow. Treatment consisted of a biological product containing a minimum of 2.7 × 10^10^ CFU/g of *B. amyloliquefaciens* QST713 at a dose of 0.935 liters/ha out of the total spray volume. Grower standard fungicide and insecticide applications were chemigated over the trial area as seeded. Blocks were 0.405 ha (1 acre) each. Harvest was conducted by harvesting 2.787 m^2^ within each block. Yield weights were evaluated and recorded in pounds and hundredweight per acre (cwt/ac).

### Sample collection.

Whole plants from the field were collected and processed to obtain bulk soil and rhizosphere samples over the three regions. The field samples were processed to obtain bulk soil from all the root surfaces by vigorous shaking, and to collect rhizosphere samples, we followed the protocol of Lundberg et al. (47) with slight modifications: roots (separated from mother tubers) were chopped into small bits and collected in a clean tube, filled with 40 ml of phosphate-buffered saline (PBS buffer), vortexed, and centrifuged to obtain a pellet. The rhizosphere pellet was stored at −80°C until genomic DNA was extracted. Samples were collected at four different time points: before planting and/or treatment (T0), 1 month after planting (T1), 2 months after planting (T2), and at harvest (T3; 5 months after planting in Grant and Sutton and 3 months after planting in White Pigeon). Samples from White Pigeon were collected for only three time points: T0, T1, and T3. From each time point, a total of 20 samples (10 treated and 10 untreated) were collected, except for White Pigeon at T0 (four treated and four untreated), T1 (12 treated and nine untreated), and T3 (four treated and four untreated) and for Grant at T0 (four treated and four untreated). A total of 84 bulk soil samples (T0 and T3) and 101 rhizosphere samples (T1 and T2) were collected. Samples were collected across different locations for each field, and the composite was submitted for analysis, in order to achieve a more homogenized sampling reducing the effect of microbial variability.

### Sample analysis.

After collection, samples were immediately sent for molecular analysis to the Biome Makers laboratory in Sacramento, CA. DNA extraction was performed with the DNeasy PowerLyzer PowerSoil kit from Qiagen. To characterize both bacterial and fungal microbial communities associated with bulk soils and rhizosphere samples, the 16S rRNA and internal transcribed spacer (ITS) marker regions were selected. Libraries were prepared following the two-step PCR Illumina protocol using custom primers amplifying the 16S rRNA V4 region and the ITS1 region described previously (48). Sequencing was conducted in an Illumina MiSeq instrument using pair-end sequencing (2 × 300 bp). For 16S, we obtained, as a mean, 51,269 reads per sample (minimum 7,253; maximum 210,185), and for ITS we obtained, as a mean, 59,183 reads per sample (minimum 8,004; maximum 348,511). See the detailed number of reads per sample in Table S7 in the supplemental material. The bioinformatic processing of reads included the merging of forward and reverse paired reads to create robust amplicons, using Vsearch (49) with minimum overlaps of 100 nucleotides and merge read sizes between 70 and 400 nucleotides. OTU clustering was performed at 97% sequence identity, followed by quality filtering through *de novo* chimera removal using the UCHIME algorithm (50). Taxonomic annotation was performed using the SINTAX algorithm (51), which uses k-mer similarity to identify the top taxonomy candidate, after which we retained results where the species level had a score of at least 0.7 bootstrap confidence. We used the SILVA database version 132 (52) and UNITE database version 7.2 (53) as taxonomic references.

10.1128/mSphere.00130-21.9 TABLE S7 Number of reads per sample. ‘*Sample identifier NCBI*’ match with the samples available in NCBI under BioProject accession code PRJNA699261. ‘*initial_reads*’ are the original number of reads. ‘*final_reads*’ are the remaining reads after applying the following filters: minimum overlap and size in merging forward and reverse paired reads, OTU clustering, quality filtering chimera removal, and taxonomic annotation compared with the reference at a minimum confidence (see a detailed description in Materials and Methods under “Sample analysis”). ‘*percentage filtered out*’ is the percentage of discarded reads in all filters [(initial − final)/initial]. Download Table S7, XLSX file, 0.02 MB. Copyright © 2021 Imam et al. 2021 Imam et al. https://creativecommons.org/licenses/by/4.0/ This content is distributed under the terms of the Creative Commons Attribution 4.0 International license.

### Alpha- and beta-diversity analysis.

Exploratory analyses of 16S and ITS OTU counts were conducted separately using the R package vegan (54). Alpha- and beta-diversity were analyzed using OTU counts. Alpha-diversity metrics (Shannon and richness) were calculated and plotted across all covariates available. Wilcoxon rank sum tests were performed to compare control and treated samples within location-time point subgroups. For beta-diversity, Kruskal’s nonmetric multidimensional scaling was performed in conjunction with Aitchison distances. Relative abundances for OTUs as well as annotations at various taxonomic levels (genera, families, etc.) were used in the analyses. Permutational multivariate analysis of variance was performed on the Aitchison distance matrix, using all possible combinations of the seed variety, location, time point (sampling time), and treatment variables.

### Differential abundance.

For all subsequent analyses, the zero counts in the data were replaced. Valid values for replacement were calculated under a Bayesian paradigm, assuming a Dirichlet prior. Nonzero values were then adjusted to maintain the overall composition (55). Differential abundance determination was carried out using the R package edgeR (56). For each OTU, the fold change attributable to the treatment across different times (e.g., T0 to T1) was calculated. This was done by conducting a hypothesis test separately for each location, measuring the fold change of a given OTU in the treatment group (from T0 to T1) versus the fold change in the control group (from T0 to T1) and then repeating the test but using times T0 to T2, and T0 to T3.

### Calculation of local network properties.

Metacommunity networks were built for 16S and ITS data separately using the methods described by Veech (57) and Ortiz-Álvarez et al. (40). In a nutshell, we first built a metacommunity network of all samples: this was done by estimating the cooccurrence and coexclusion that would occur solely by chance for all possible OTU pairs, given the data. We selected OTU pairs that occurred significantly more than expected by chance to create the cooccurrence networks. As an initial filter, for bacteria, we retained OTUs that were detected in at least 30% of the entire data set, and 10% for fungal communities. This is due to the disproportionate number of unique OTUs detected in 16S versus ITS soil sequencing. To keep the overall size of the data manageable, we limited the number of selected OTUs to 4,000 with a maximum of 10 million possible significant pairs. This resulted in metacommunity networks consisting of 3,339 nodes for bacteria (19.4% of the total 17,241 bacterial OTUs) and 447 nodes for fungi (26.3% of the total 1,702 fungal OTUs), which on average captured 92.11% of the bacterial abundance and 98.62% of the fungal abundance of the samples in the study.

Similarly, those that occurred significantly fewer times than expected by chance constituted the coexclusion network. Local networks (single-sample level) were calculated by subsetting the metacommunity network for OTU pairs detected in each sample and estimating a local network. The R package igraph was used to calculate network properties: modularity, transitivity, and proportion of coexclusions and cooccurrences in relation to the total number of combinations among all OTUs in a sample (58). An adequate description of the ecological meaning of the different network properties calculated in this work can be found in the review work of Proulx et al. (59). Network properties were compared using a linear model. Using the network property as outcome, hypothesis tests were performed to compare time point differences in treated versus control samples (analogous to the approach used for investigating differential abundances).

### Yield model.

Yield data were first explored using medians and interquartile ranges (IQRs). Wilcoxon rank sum tests were performed on these yield data. The OTU counts were transformed using the centered log-ratio (CLR) transformation. CLR-transformed 16S and ITS data were jointly projected onto 70 principal components. Yield was modeled as the outcome of these 70 principal components, along with fungal and bacterial network properties, treatment, soil type (to distinguish between bulk and rhizosphere soils), and a variable that encompasses seed variety and geography, using a probability forest as described by Malley et al. (60). Since the yield is a constant variable for all time points within a block, the yield was converted to a categorical variable (≤30 t/ha, >30 t/ha). The threshold for this division, 30 tonnes, was set at a zero-probability density point for the bimodal distribution of yield. We used a total of 104 samples (all T0 through T2 samples in the study for which we had yield data) and split them into training (*n* = 78) and test (*n* = 26) sets. Variable importance for each variable in the model was calculated using the Gini index. As a sensitivity test, probability forests were fit for a three-way split of the yield variable, and variable importances were compared. Among the 70 principal components of the microbiome included in the model, the ones with the highest importance in the probability forest were selected for further analysis. The loadings of these principal components were clustered using an unsupervised hierarchical clustering algorithm to visualize some of the most influential OTUs’ impact on these principal components.

### Data availability.

Raw files for bacterial and fungal amplicons for each sample are available in NCBI under BioProject accession code PRJNA699261.
