# Supplementary figures and images for: Local Network Properties of Soil and Rhizosphere Microbial Communities in Potato Plantations Treated with a Biological Product Are Important Predictors of Crop Yield
Source: mSphere. 2021 Aug 11;6(4):e00130-21. doi: 10.1128/mSphere.00130-21 (PMC8386434; doi:10.1128/mSphere.00130-21)

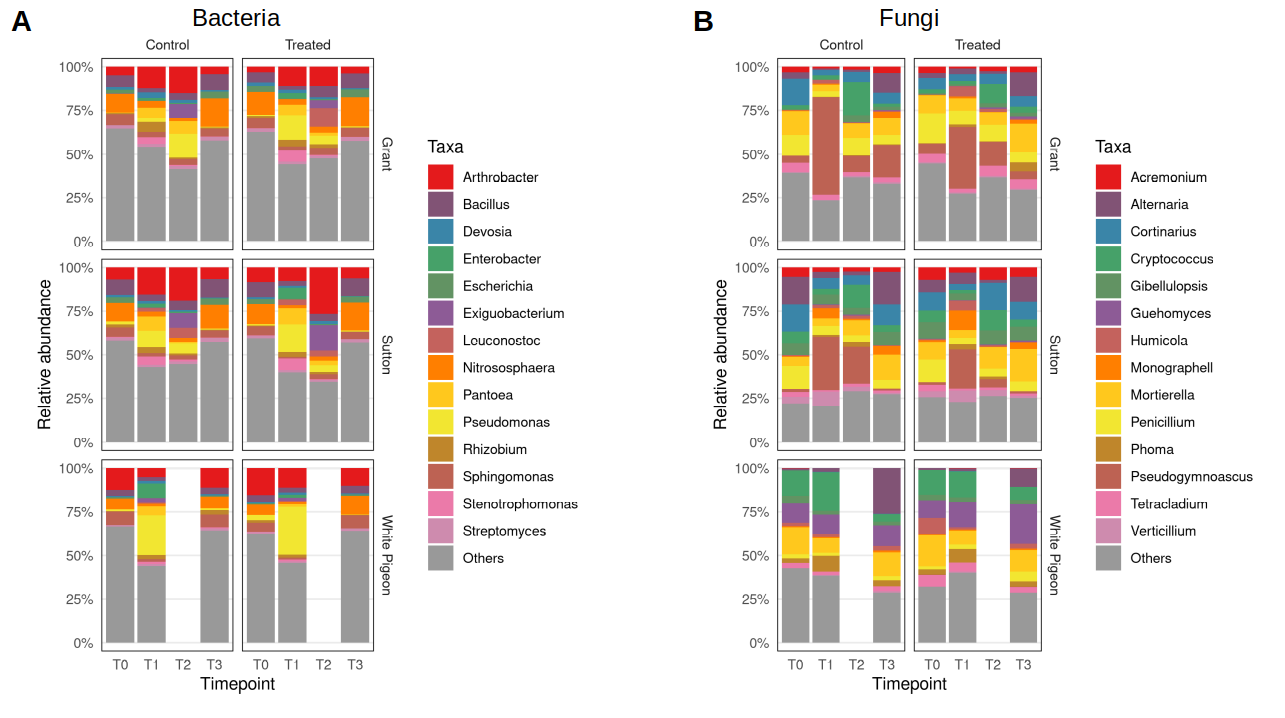

Supplement: FIG S1 [file msphere.00130-21-sf001.tif]

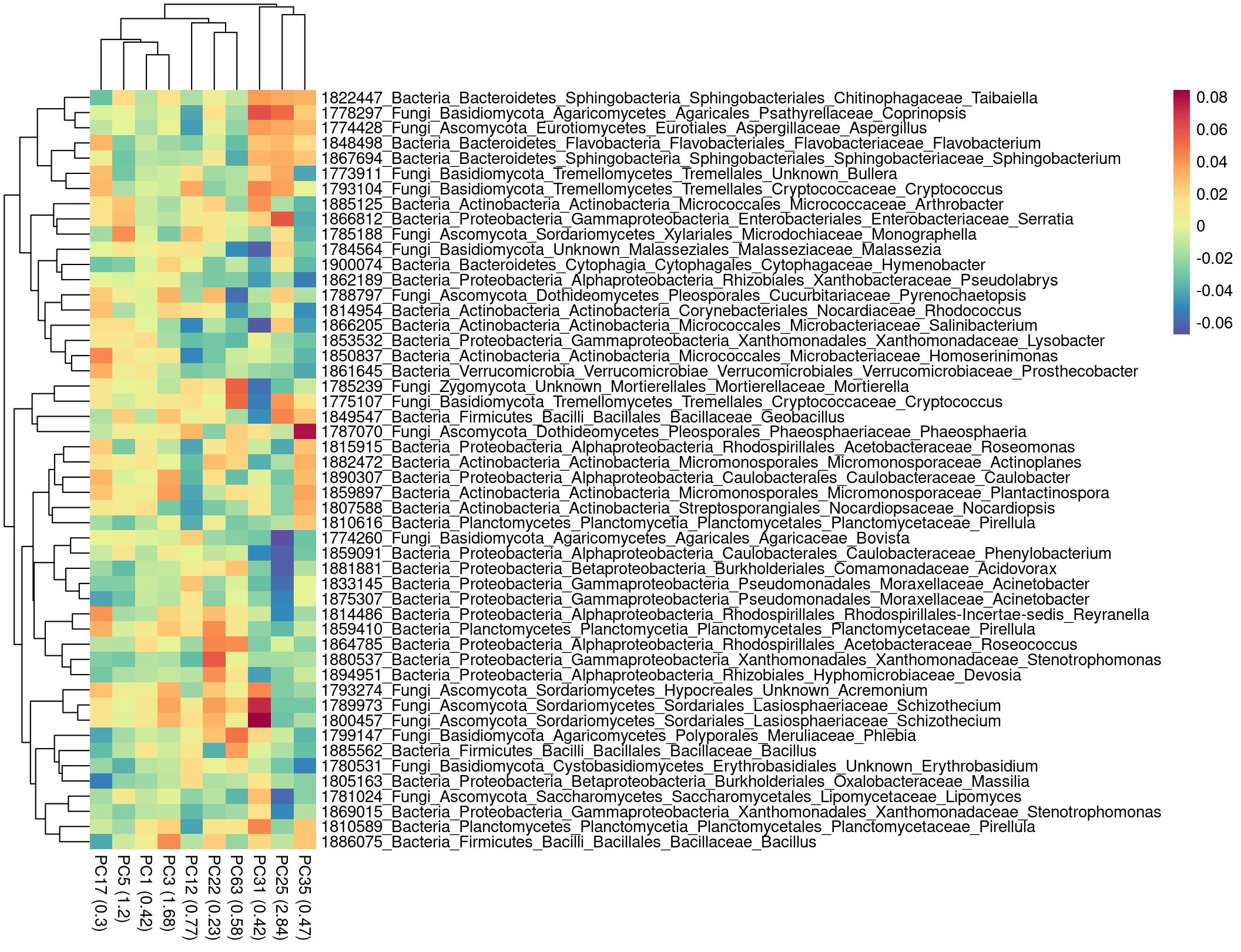

Supplement: FIG S2 [file msphere.00130-21-sf002.tif]
